# Promoting Geropsychology: A Memorandum for Research, Policies, Education Programs, and Practices for Healthy Aging

**DOI:** 10.3390/ijerph21091172

**Published:** 2024-09-03

**Authors:** Andrea Bosco, Anna Consiglio, Manuela Nicoletta Di Masi, Antonella Lopez

**Affiliations:** 1 Department of Educational Sciences, Psychology, Communication, University of Bari, 70122 Bari, Italy; andrea.bosco@uniba.it (A.B.); anna.consiglio@uniba.it (A.C.); 2 IBiSS Società Cooperativa Sociale, 70018 Rutigliano, Italy; presidente@ibiss.org; 3 Faculty of Law, Giustino Fortunato University, 82100 Benevento, Italy

**Keywords:** geropsychology, policy, assessment, training programs, clinical psychology, active aging, cognitive reserve, successful aging

## Abstract

Background: This viewpoint paper reports the state of the art at a global level on research, practice and assessment, policies, and training in the clinical psychology of aging and, more specifically, in geropsychology. The main sources of information were as follows: (1) the most recent reviews of the literature available in the scientific literature; (2) the resources on the internet referable to professional and academic associations dealing with the topic; and (3) the laws, policy initiatives, and funded programs that are aimed at the diffusion and applications of mental health in aging. Methods: The present study aims to provide an updated and comprehensive memorandum highlighting the importance of prioritizing mental health in older adults. It seeks to promote health in general and disease prevention strategies, ensuring equitable access to mental health services integrated into primary care and designed for aging. This paper also aims to shed light on the slow development process and lack of consolidation in the adaptation of academic training at master’s and doctoral levels in most developed countries, despite the long-declared importance of enhancing resources for the promotion of geropsychology. Results: The results of the present study are patchy. Although the importance of enhancing resources for the promotion of geropsychology has long been declared, the development process seems very slow, and the adaptation of academic training at master’s and doctoral levels in most developed countries—those that, for demographic reasons and attitudes, should be more sensitive to the issue, does not yet seem to have consolidated. Conclusions: Collaboration among diverse professionals is crucial for providing integrated and comprehensive care to older adults that addresses their physical, psychological, and social needs.

## 1. Introduction

There is a stage of life in which individuals reach full physical and mental maturity. During this phase, the organism—the harmonious integration of body and mind—thrives [1]. It adeptly responds to stress, maintains resilience even in the face of exhausting work, and fulfills the multifaceted roles of family care. This includes nurturing children, supporting their physical and intellectual growth, and occasionally extending their support to the broader community [2]. Amidst the hustle, individuals also carve out moments for leisure activities, finding solace and satisfaction therein. However, as time progresses, the activities undergo a natural slowdown that corresponds to that of all the lively functions. Additionally, there is a subtle but perceptible decline in the capacity to cope with environmental demands [3]. In a word, people age.

It is a widely held belief that there is a general deterioration in physical functioning in aging. Each person is usually aware of this. Scarcer are the scientific contributions on literacy related to mental health in aging [4] and especially related to how the latter interacts intimately with physical health [5]. Nonetheless, cognitive impairments associated with aging are well-recognized [6]. Dementia still represents the main topic that, in the collective imagery, is experienced as a frightening and deeply concerning condition associated with memory loss, confusion, and a gradual decline in cognitive abilities [7]. The topic is so relevant that a paramount number of tests have been developed to measure the phenomenon [8]. The progression of dementia is often portrayed as relentless and irreversible, contributing to the sense of hopelessness and despair associated with the condition. There is a pervasive fear surrounding the prospect of losing one’s mental faculties and autonomy, as well as the burden it places on caregivers and family members. On the other side, recognizing cognitive efficiency as a pivotal aspect of mental health underscores the relevance and urgency of this discourse. According to the WHO [9], the number of people over the age of 60 is expected to double by 2050, intensifying the challenges associated with age-related diseases, including dementia and other neurodegenerative conditions. This increase not only poses a significant public health challenge but also presents a major economic burden for countries globally. Given these alarming projections and the associated economic impact, any discipline or intervention that can mitigate the effects of pathological aging is not only welcome but essential for sustainable healthcare systems and the well-being of aging populations. Public health policies must prioritize preventive measures, such as promoting physical activity, to curb the growing burden of age-related diseases.

According to Livingston’s comprehensive review [10], numerous additional factors intersect with cognitive efficiency to influence psychological well-being, collectively distinguishing between successful aging and frailty, which signifies a less favorable aging trajectory. These factors not only affect cognitive health but also contribute to the overall aging process, potentially accelerating cognitive decline and increasing the likelihood of developing dementia.

One such factor is cognitive reserve, which refers to the mind’s resilience to neuropathological damage. It should be distinguished from brain reserve, a sort of physical trait. Individuals with a higher initial brain capacity can endure more neurological decline before experiencing impairment [11,12].

Individuals with higher cognitive reserve, often developed through lifelong learning and mental engagement, can better maintain cognitive functions despite age-related brain changes. This concept underscores the value of education, complex occupational tasks, and engaging leisure activities in building cognitive reserve, which can help delay the onset of cognitive decline [13,14,15,16,17,18,19].

Moreover, physical activity is one of the most well-established contributors to healthy aging. Regular exercise offers numerous benefits that are crucial for maintaining physical health, cognitive function, and emotional well-being. Research has shown that regular physical activity can improve brain plasticity, reduce the risk of dementia, and enhance cognitive functions such as memory, attention, and executive functioning [20]. Additionally, social isolation and loneliness are significant risk factors for poor health outcomes in older adults. Therefore, addressing loneliness is essential for promoting healthy aging and mental well-being. Older adults who maintain strong social ties are less likely to experience depression, anxiety, and cognitive decline. Engaging in community activities, joining clubs, and staying in touch with family and friends can provide emotional support and a sense of belonging [21].

Additionally, in the digital age, digital affinity—or the capacity to engage with and utilize digital technologies—has become increasingly relevant in the psychology of aging. Older adults who maintain or develop digital skills can benefit from enhanced cognitive stimulation, social connectivity, and access to information. Digital literacy programs for the elderly can play a crucial role in reducing the digital divide and promoting mental health and cognitive resilience. Engaging with technology not only supports cognitive functions but also contributes to a sense of competence and autonomy in an ever-evolving digital world [22,23].

The limited understanding of mental health’s role in aging can be attributed not only to cultural factors such as stigma [24], lack of knowledge [25], and perceived insignificance [26] but also to deficient media coverage and the minimal impact of welfare services in disseminating widespread awareness about the critical role of psychological well-being in overall health.

Thus, we aimed to document and disseminate research and practice related to the topic to a broader audience. In pursuit of this goal, we acknowledged the importance of academic and professional training in the field. We then briefly explored informative resources provided by leading Scientific Societies worldwide in the Clinical Psychology of Aging. Our long-term aim is to assess the potential feasibility and sustainability of the clinical psychology of aging services and their potential long-term impact on improving public health.

There is a specialized field within psychology that focuses on addressing the mental health and emotional well-being of older adults, known as geropsychology [27]. It involves the study, assessment, diagnosis, and treatment of psychological issues and disorders that commonly affect elderly individuals. It applies the knowledge and methods of psychology to understanding and helping older persons and their families to maintain well-being, overcome problems, and achieve maximum potential during later life. Geropsychology appreciates the wide diversity among older adults, the complex ethical issues that can arise in geriatric practice, and the importance of interdisciplinary models of care. Geropsychology is also known as the clinical psychology of aging. Both involve understanding the unique challenges and complexities of aging and tailoring interventions to meet the needs of this population [28]. To clarify the use of the terms, The American Psychological Association (APA) [29] does not officially recognize distinct subfields called “geropsychology” and “clinical psychology of aging”. Rather, these terms are often used interchangeably to refer to the psychological study of aging and its associated processes. However, some professionals and organizations may make distinctions between the two terms based on subtle differences in focus or emphasis. For example, geropsychology may sometimes be used to describe the application of psychological principles and practices specifically within clinical settings, such as providing mental health services to older adults or conducting research on age-related cognitive decline and interventions [30]. On the other hand, the clinical psychology of aging may have a broader connotation, encompassing not only clinical aspects but also research into the psychological, social, and biological aspects of aging across the lifespan. It may include studying topics such as successful aging, cognitive development in later life, social relationships, and societal attitudes toward aging. So, while there may be some nuanced differences in how these terms are used in practice, they both generally refer to the study of aging from a psychological perspective, taking into account assessment (clinical interviewing, behavioral and environmental observation, self-report measures, cognitive and functional performance testing), intervention (utilization, adaptation, and integration of evidence-based psychological interventions, health promotion), and consultation (with families, professionals, service agencies, communities, legal systems), and the distinction between them may vary depending on context and individual interpretation rather than being formally defined by the APA. It is important to underline that, to the best of our knowledge, no review was present differentiating the two terms “clinical psychology of aging” and “geropsychology”.

In the following viewpoint paper, deemed to combine the critical analysis of a perspective with the scholarly rigor expected in reviews, offering a focused and independent perspective on current scientific issues [31], the term geropsychology will be used as an umbrella term to encompass the psychological research and applications concerning the aging processes.

## 2. Geropsychology: Laws and Policies

Geropsychologists need to keep abreast of laws and policies to ensure compliance and to be able to provide ethical and effective mental health care to older adults. Ongoing advocacy efforts are also essential to address gaps in existing laws and promote policies that support the well-being and dignity of elderly individuals.

Geropsychologists should stay informed about laws and policies while advocating for improvements to ensure ethical, effective care and support for the well-being of older adults, especially in the face of global aging challenges that influence public policies, legislation, and mental health services.

### 2.1. Responsibilities and Advocacy in Elder Care

For many years, the impact of the progressive aging of the population on the global demographic structure has long been the subject of attention by regulators and policymakers at the national and international levels [32].

This consideration becomes even more compelling when we consider that in various parts of the Western world, the working population is expected to decline progressively because of the advanced stage of demographic transition and that it is estimated that two-thirds of the world’s elderly population will live in developing countries (WHO) [33].

The implications and possible scenarios of these trends for welfare systems, in particular pension systems, for intergenerational solidarity, and for the cost of health care—especially long-term care (LTC)—are evoked at a lexical level in the conceptualization of the issue, in terms of conflict, bomb, war [34,35], which has implications for the development of public policies and legislation. The profound physical, psychological, and social changes associated with the aging of the world’s population, therefore, represent a crucial challenge for international institutions and bodies, which are called upon to weave a network of decisions and actions that allocate values [36] (p. 130) against the background of the results and directions of scientific research. These policy documents are intended to have a decisive impact on national policymaking by outlining a broad, coherent framework of regulatory and policy production calibrated to individual territorial conditions.

The review by Doron and Mewhinney [37] and the analysis by Plath [38] trace a complex and differentiated path of international legislation and policymaking on aging due to the plurality of actors and their respective levels, governance, territorial scale, and logics of action [39], that underpin public policies.

### 2.2. Evolution of International Aging Policies and Legislation

Undoubtedly, the development of strategies on the subject at global, national, and local levels began after the Second World War, in the same years in which gerontology developed its first interdisciplinary approach to the subject with the studies of Shock [40].

In the broad international framework of documents and instruments on aging and the human rights of older people, it is the United Nations (UN) Assembly that has developed the ethical–legal reference framework, with Article 25 of the Universal Declaration of Human Rights (1948), together with one of its three implementing instruments, the International Covenant on Economic, Social, and Cultural Rights (1966). They affirm the right of everyone, including the elderly, to health care and social services with an adequate standard.

During these years, the thinking of non-governmental organizations was also active, such as the International Labour Organization (ILO) Convention on Social Security (Minimum Standards) (1952) and the ILO Convention on Disability, Old Age, and Survivors’ Benefits (1967), which established the universally recognized standards in the field of social security, including for older workers.

On the geopolitical side, if the United States (American Declaration of the Rights and Duties of Man, 1948; American Convention on Human Rights “Pact of San Jose, Costa Rica”, 1969) and the European Community (European Convention on Human Rights, 1950; European Interim Agreement on Social Security Schemes Relating to Old Age, Invalidity and Survivors, 1953), can be seen as forerunners, while in the rest of the world, public policy reflection became established between the 1980s—Africa—and 1990s—Asia and the Arab–Islamic world [41].

The time gap in regulatory production between the different continents is significant in terms of legal conceptualizations and theoretical models on the aging of references. In the first phase—North American and European—the focus is on the human rights and the dignity of the person, while in the Afro-Asian area, there are also references to health in general, to social life, and to raising awareness among health workers.

In fact, between the 1960s and the 1980s, the functionalist vision of old age of Cumming and Henry [42], in which occupational and social retirement is presented as an inevitable phase of human existence—to leave room for conscious detachment from life and to allow for generational change in terms of productivity and efficiency—is being replaced by models that emphasize the role of psychophysical well-being (SA) and socio-economic integration (AA) in remaining active into old age [43,44,45].

However, it should be emphasized that the establishment of legal principles was not followed by a simultaneous parallel planning and strategic intervention activity.

It took 20 years from the declaratory commitment of Resolution A/C.3/213 of 1948, in which Argentina proposed to the UN General Assembly a set of ten rights to protect older persons—assistance, shelter, food, privacy, care of physical health, care of moral health, recreation, work, stability, respect—to the first World Assembly on Ageing in Vienna, Austria in 1982 [46].

The Vienna Plan of Action is the first international instrument to address the challenges of aging through recommendations to Member States in seven fundamental areas (UN, 1982), including three areas of intervention for policymakers: data collection and analysis, education and training, and research. Aging is presented as a “long-life process”, which also includes the psychological dimension [47].

The International Federation on Ageing (IFA) Declaration of Rights and Responsibilities of Older Persons (1990) summarizes the fundamental core of the regulatory and policy orientations developed to date: independence—in terms of adequacy to the standard of living of the population; participation—involvement of older people as stakeholders; health and social services at the systemic-family level; full fulfillment of the person; dignity and responsibility—in terms of commitment to active, competent and meaningful aging.

The document explicitly refers to the right to mental health and introduces the tool of prevention and rehabilitation in order to reduce public health expenditure.

In 1997, 15 years after the first World Assembly on Ageing, the global results appeared to be uneven, mainly due to the allocation of resources, the low priority of aging in political agendas, and the inadequacy of infrastructures [48].

However, in the years leading up to the Second World Assembly on Ageing, on the way to Madrid in 2002, some important achievements can be noted: the creation of the International Institute on Ageing (1988) and the African Society of Gerontology (1989); the proclamation of 1 October as International Older Persons’ Day (1990); the adoption of the UN Principles for Older Persons (1991).

The year 1995 was fundamental for the systemic implementation of mental health [49]: in the UN’s General Comment 6 on the Economic, Social and Cultural Rights of Older Persons, the right to physical health is accompanied by the right to mental health, and the Healthy Ageing paradigm is adopted by the WHO, which understands aging as a universal process from a biopsychosocial perspective in order to maintain functional independence and the absence of disease [50].

In preparation for the International Year of Older Persons in 1999 (UN, 1999), the UN proposed a conceptual framework for a society ‘for all ages’, divided into four dimensions, with psychological well-being playing a role in the dimension of lifelong individual development and health care in the dimension of aging and development [41].

Subsequently, with the launch of the International Plan of Action on Ageing in 2002, the WHO committed itself to work with other intergovernmental organizations, non-governmental organizations (NGOs), and the academic sector to develop a global framework for research on aging.

The WHO proposal, “Active ageing: a policy framework” [51], is a conceptual and operational platform for legislative initiatives and policy strategies that can unite the interests of the main stakeholders: citizens, non-governmental organizations, economic interests, governments, and administrative bodies.

The document considers mental health, along with physical well-being and social connectedness, as a crucial factor contributing to the quality of life of older people: mental illnesses such as dementia and depression are among the main causes of disability in older people, affecting their autonomy and independence.

### 2.3. Outcomes and Challenges of Strategic Aging Planning

On the basis of these assumptions, it is considered necessary to include mental health services in the multicomponent model of long-term care when designing an integrated, coordinated, and economically advantageous care system, with particular reference to the phenomenon of under-diagnosis of mental disorders, mental illness, and suicide in older people [51].

With regard to three pillars of active aging—health, participation, and security—an action to inform and combat stereotypes about mental health is proposed to institutional actors, promoting a positive vision of mental health and encouraging an integrated approach within mental health services, from prevention to treatment of mental pathologies, from rehabilitation to reintegration into the communities to which patients belong. Attention is also given to the home treatment of dementia, neurological disorders, and intellectual disabilities.

The adoption of this model in numerous strategic plans, as has been said, finds a global systematization and legitimation in the Madrid International Plan of Action on Aging (MIPAA) [52], which, although not boasting binding legal efficacy towards member countries, represents the reference matrix for interventions on aging, at macro, meso, and micro levels, in different areas of the planet.

Founded on the principle of a society for all ages, the MIPAA 2002 plan [53] is divided into objectives and actions that each country must take, considering the approach to aging from a development perspective—integrating the elderly into strategic documents and policymaking—and the notion of intergenerational solidarity. Three priorities are present: (1) older persons and development; (2) advancing health and well-being into old age; and (3) ensuring, enabling, and supportive environments. The development of an integrated mental health service, which operates in the following areas, has also been introduced: Prevention and Early Detection, Treatment and Care, Supporting Independence, Reintegration and Community Services, Dignity and Care Facilities, Public Awareness; Care in Long-Term Facilities; Professional Development [53].

Through the system of five-year review processes, it is possible to monitor the progress of the plan through the reports that the individual territorial areas submit at the end of each implementation cycle, drawn up based on the guidelines prepared by the Standing Working Group on Aging.

In the third cycle (MIPAA+15), 2017, there was a strategic alignment with the Global Agenda for Sustainable Development and the Sustainable Development Goals (SDGs) to be achieved by 2030. The implementation at a national level, through the adoption of “National Strategies for Sustainable Development”, is no longer limited to the economic dimension but combined with the creation of a fundamental pillar, that of inclusion, in the Decade of Healthy Ageing [54].

Mental health is central to SDG 3 because it focuses on health and well-being, but it is also linked to other SDGs because of the relationship between mental health and poverty (SDG 1) and the reduction in inequalities (SDG 10), among other SDGs.

On the fourth review and evaluation cycle (2017–2022), which coincides with the 20th anniversary of the implementation of the MIPAA plan, the impact of the COVID-19 pandemic was considered in light of the policy brief: The Impact of COVID-19 on Older People, published in 2020.

At a global level, significant critical issues have been identified, particularly in the areas of health (vaccination policies, discretionary choices about health service targets/levels of care, and available therapies) and human rights (limitations due to social segregation), with the consequent exacerbation of the phenomena of ageism, discrimination, and stigmatization of older people [55].

The need to promote gerontology and geriatric skills in tertiary studies [56] and the attention that MIPAA gives to the problem of training medical and health professionals in gerontological skills and the careers of gerontologists [54] indicate the urgency of creating frameworks and operational lines aimed at generalist medicine within national health systems.

Among the many overall results of MIPAA, it is possible to identify key strategic lines and legislative interventions, starting with Western Europe and North America. In fact, since 2006, the European Union has expressed a political direction aimed at supporting aging as an opportunity for society, promoting health through the paradigm of active aging, and intervening in sustainable welfare systems for the demands of the elderly population, with a particular focus on policies related to long-term care [57]. In some countries of the United Nations Economic Commission for Europe (ECE) region [58], the foundations of geriatric health care were laid during the fourth cycle of MIPAA/RIS (Regional Implementation Strategy) implementation (ARM, AZE, BLR, KAZ), while in others existing geriatric health services were developed through new regulations, protocols, or manuals (MDA, TJK) and additional professional education and training (RUS, TJK). Other countries in the region have piloted (RUS), introduced (ALB, BGR, TJK), updated (FRA, NLD), or are in the process of reforming (AUT, TJK) their comprehensive policies to meet the long-term health and long-term health and social care needs of their aging populations (AUT, ESP, EST, GRC, LUX). In the UK, the National Health Service (NHS) Long Term Plan and the NHS Mental Health Implementation Plan 2019/20–2023/24 addressed the NHS to provide consistent access to mental health care for older adults with functional needs (i.e., depression, anxiety, and severe mental illness). Investment in higher education in geriatrics and dementia is significant in the UK and the Nordic countries and, to a lesser extent, in Germany, Italy, and Turkey [59].

The picture that emerges from the reports of the MIPAA regions highlights, on the one hand, the growing attention paid to the issue of aging in political strategies and government agendas, with results that are in some cases highly significant for integrated approaches to care [60], and, on the other hand, doubts about the penetration of global plans with limited soft law characteristics, expressing recommendations to achieve broad socio-economic objectives, with particular attention to spending cuts published in relation to aging [52].

In the United States [61], even in the presence of legislation aimed at supporting caregivers, funding for long-term services and support is targeted at an older population that generally avoids institutionalization. The implementation of a national network of services for older people to connect the elderly to local actors has proven difficult, and the virtual service provision mechanism is unreliable. The practice of sharing data collection and information on abuse and fraud has proved effective and contributes to the monitoring of government prevention policies, highlighting the importance of reliable and valid tools to measure interventions.

Although policies have made the use of screening and outpatient care more accessible, access to mental health care for the elderly with basic health insurance remains controversial because of limited adherence due to the structure of the US health care system, the salary of health care professionals, and the high cost of psychiatric medications.

The situation in Canada is more complex, with widespread burnout among long-term care workers and difficulties in accessing primary care in remote areas [62].

The national reviews presented at the High-Level Political Forum on Sustainable Development for the period 2016–2019 confirm the picture of a fragile elderly population that places a burden on social services, pensions, and the health system and limits economic growth in the long term, according to a vision characteristic of neoliberalism [63].

There are clear challenges in the provision of mental health care in Africa, ranging from gaps in government mental health policies—given the difficult coexistence of religious healers and mental health care provision—funding challenges, inadequate mental health facilities, lack of human resources, and poor training and development programs for health professionals [64].

In Oceania, despite the acceptance of MIPAA requirements by policymakers, there is limited impact on national and regional policies, with disparities in access to care that disadvantage ethnic minorities. In Australia, following the Royal Commission into Aged Care Quality and Safety 2018–2021, the Older Peoples Mental Health (OPMH) provides community-based clinical mental health care, guided by the New South Wales (NSW) Service Plan for Older People’s Mental Health Services 2017–2027 [65].

In the Asian region, mental health issues have not been widely included in national priorities. In the Republic of Korea, the model of Community Dementia Reassurance Centres is spreading, where residents and people with dementia can receive diagnostic services and counseling, as well as participate in activities to improve their cognitive skills [66]. In China, the homogeneity of mental health services across geographical areas has increased with the start of the health system reform in 2009. Numerous policy interventions and the enactment of the Mental Health Law have been conducive to improving the mental health service system, as well as staff service capacity and service models [67]. In India, the National Mental Health Policy (NMH) introduced in 2014 and the Mental Healthcare Act (MHCA) of 2017 have highlighted the heterogeneity of the healthcare system across the country and the influence of religious beliefs on evidence-based medicine. The recent intervention programs developed with the National Mental Health Program (NMHP) and the District Mental Health Program (DMHP) show positive results in terms of community involvement and broadening the reference target [68,69,70,71].

With the proclamation of the Decade of Healthy Ageing (2021–2030), the UN has given WHO the mandate to lead the implementation of the Decade itself, with global collaborative action to improve the lives of older people, their families, and the communities in which they live, through policies and systems strategies implemented by national and international partners [72].

The Healthy Ageing conceptualization proposal defines aging as a process of multi-sectoral changes that enable well-being in old age [73]; divided into three components—environment, functional capacity and intrinsic capacity—it allows the alignment of healthy aging with the United Nations Sustainable Development Goals (SDGs) [74] and the connection with other analytical documents and policies—the World Report on Ageing and Health (2015) and the Global Strategy and Action Plan on Ageing and Health (2017)—and other key global issues, such as the Global Action Plan on the Public Health Response to Dementia 2017–2025 [75,76].

In the four areas of intervention, combating ageism, age-friendly environments, person-centered integrated care and primary health care, and long-term care, mental health is explicitly included in combating ageism, in the training of health professionals, in prevention, in primary and long-term care and in health emergencies [51,77,78].

Although the beginning of the Decade was marked by many challenges, such as the COVID-19 pandemic, the global economic crisis, and international conflicts, Member States have made much progress [79].

For a fruitful continuation of the Decade, it is even more necessary to promote interaction between researchers, policymakers, and other stakeholders who can synergistically influence the uptake of research findings [80] and the involvement of the older population itself in the process of enabling everyone to age well [81,82].

## 3. Geropsychology in the Literature Reviews

Various academic journals in the field of psychology, gerontology, or related disciplines have been published focusing on the foundations of geropsychology and the related topics, as well as the clinical psychology of aging.

As already stated, the American Psychological Association (APA) represents the forefront reference for the subject matter. In fact, the first issue worthy of note of the APA journal Clinical Psychology: Science and Practice in 2022 is completely dedicated to the foundational knowledge competency in geropsychology.

This could include topics such as understanding the psychological aspects of aging, assessment, and diagnosis of age-related mental health issues; evidence-based interventions for older adults; ethical considerations in working with older populations; cultural competence in geropsychology practice; and interdisciplinary collaboration in geriatric care. The special issue may feature research articles, review papers, and perspectives from experts in the field to provide a comprehensive overview of foundational knowledge and competencies required for effective practice in geropsychology.

The topics were addressed through five papers of high caliber and potential impact. The first by Garrison-Diehn and colleagues [83] focuses on attitudes toward older adults. This paper extensively addresses the topic of ageism: the prejudice (overt and covert) and discriminatory acts based on a person’s age. The second, by Woodhead and Yochim [84], discusses aging from the perspective of developmental psychology. It covers demographics, individual differences in the aging process; contemporary theories of aging, the role of physical health; and social, personal, and emotional development affecting self-perception and well-being. All of these issues are within the context that places issues reported by the elderly within the framework of aging changes. The authors conclude that enhancing knowledge about adult development and aging may facilitate case comprehension and intervention planning. Additionally, the paper describes the development of the Pikes Peak Model of Training in professional geropsychology, which is likely the most advanced and well-defined training approach on the topic, more than 15 years after its establishment. The third paper by Jacobs and Bamonti [85] is more directly oriented towards the practice of clinical psychology and all the implications it has with specific aspects of the elderly person’s condition. Complications due to medical illnesses, functional impairments, and late-life stressors are discussed. Sensory, cognitive, and functional changes related to age or physical illnesses necessitate intervention adaptations and a global, holistic care approach to minimize maladjustment and promote physical, psychological, and interpersonal well-being. The fourth paper by Mast and colleagues [86] is devoted to assessment and evaluation methods. A significant portion of psychologists report working with older adults with relatively poor formal training. The authors focus on key principles of geropsychological assessment: theories; essential research findings on cognition, emotion, personality, and interpersonal relationships; measurement problems; and tests. Finally, Lind and colleagues [87] approach the topic of intervention, consultation, and services in clinical geropsychology. They review psychological interventions specifically tailored or adapted to older adults and address medical issues impacting the quality of psychological services. The role of interdisciplinary collaboration in providing care is discussed, as well as primary prevention services promoting healthy lifestyles and preventing disease. Finally, they address best practices regarding administrative, ethical, and legal issues, including informed consent, confidentiality, end-of-life issues, conflicts of interest, and elder abuse and neglect. These five reviews are further enriched by a series of commentaries from prominent figures on the topic.

Currently, 34 reviews have been published from 1970 to the present in the field of geropsychology (following the PRISMA statement, including original articles written in English on PubMed, Scopus, and Web of Science, on 25 March 2024). Starting from the seminal work of Botwinick [88], in which the author examines research findings and theoretical perspectives relevant to geropsychology from 1963 up to the year 1970, including studies on age-related changes in memory, intelligence, personality, and adaptation to life transitions in old age. Additionally, it might discuss the implications of these findings for understanding aging processes and for developing interventions to support older adults. In the 90s, researchers focused their attention on the evolving landscape of psychotherapy concerning older adults [89], on strategies for incorporating geropsychiatric content into nursing education curricula [90], and on the design considerations and construction demands associated with creating housing options tailored to the needs of older adults [91]. The first decade of the 2000s, and in particular 2005, saw a spike in scientific production [92,93,94,95,96,97,98,99,100,101,102,103,104,105,106,107,108,109,110]. The research contributes to the understanding of geropsychology and psychogeriatrics, addressing various aspects such as research, education, and clinical practice, thereby offering valuable insights and guidance for professionals working with older adults. The second decade has seen the publication of several papers [111,112,113,114,115,116] that collectively highlight the importance of addressing mental health issues in older adults, considering cognitive, emotional, social, and cultural factors. They emphasize the need for tailored interventions and ethical considerations in geropsychological practice, focusing on research, clinical practice, and training programs specific to geropsychology within different countries and cultural contexts. The last 4 years of research seem to have focused their attention on various aspects of geropsychology, addressing the psychological well-being and mental health needs of older adults. Each paper offers insights into different dimensions of this field, such as the supervision of trainees, psychological flexibility, cultural considerations, and the integration of spirituality into psychological interventions for older adults [117,118,119,120,121].

## 4. Geropsychology through Self-Presentations and Resources Made Available by Scientific Societies

After the formal recognition of geropsychology as a distinct specialty in psychology, various global and national organizations developed guidelines, training, and research efforts to address the unique mental health needs of older adults, though the field’s development and emphasis vary significantly across regions.

In 2010, geropsychology was established as a distinct specialty within professional psychology. The urgency of expanding and enhancing the workforce in this field was underscored in 2012 by the Institute of Medicine’s study titled “*The Mental Health and Substance Use Workforce for Older Adults: In Whose Hands*?” This emphasized the need to address the challenges posed by our rapidly aging and evolving population [122,123]. Subsequently, in 2014, the American Psychological Association updated the Guidelines for Psychological Practice with Older Adults, officially recognizing geropsychology as a specialty [124]. Additionally, the American Board of Professional Psychology acknowledged geropsychology as a distinct specialty during the same year [125].

According to the self-presentation of the American Psychological Association [126], division 12: Society of Clinical Psychology [127], Clinical Psychology involves research, teaching, and services relevant to the applications of principles, methods, and procedures for understanding, predicting, and alleviating intellectual, emotional, biological, psychological, social and behavioral maladjustment, disability, and discomfort, applied to a wide range of client populations. In theory, training, and practice, Clinical Psychology strives to recognize the importance of diversity and strives to understand the roles of gender, culture, ethnicity, race, sexual orientation, and other dimensions of diversity. Hence, diversity due to age is not explicitly mentioned in the text. Instead, actions to predict and reduce the effects of biological disorders as well as mental disorders are mentioned as a goal.

Nonetheless, within division 12, there is Section II, Clinical Geropsychology of the Society for Clinical Geropsychology [128], which is presented by the following statements: The vision of the Society of Clinical Geropsychology is to foster the mental health and wellness of older adults through science, practice, education and advocacy and to advance the field of professional geropsychology. Our purpose is to promote the general objectives of the American Psychological Association and Division 12 [129]; to support and to encourage the evolution and development of the subspecialty of clinical geropsychology in both its scientific and professional aspects; to increase scientific understanding of the mental health of older adults; to promote the development of models for the delivery of psychological services to older adults, as well as other ways of enhancing the welfare and mental health of older adults; to foster collaboration and the sharing of information among clinical geropsychologists; and to increase the quality and availability of training opportunities in clinical geropsychology.

Thus, there was a clear recognition by the largest and most important worldwide scientific association in psychology of the need to develop a discipline, a subspecialty, that is specifically geared toward clinical psychology for older adults. Coherently, in 2003, the APA produced the Guidelines for Psychological Practice with Older Adults, updated in 2014 and further in 2024 [130,131]. The Guidelines represent the planet-wide cornerstone of any psychological care professional who intends to work with older persons.

Analyzing the European context from the point of view of Scientific Societies is not trivial. The reason is that there is no equivalent of the American APA, and there are mostly national or international societies that are unified within federations. To the best of our knowledge, the most important and recognized is the European Federation of Psychologists Associations (EFPA) [132]. Within its organization, a Standing Committee entitled Geropsychology is present [133]. In the initial presentation, after the usual description of European demography and the increasing massive presence of elderly people, we read the following: “It is a challenge for Psychology and especially Geropsychology to provide sound research-based knowledge about the diverse psychological processes underlying human ageing as well as expertise about training, education and interventions that will help to promote quality of living and subjective well-being both at the individual as well as the social level”. The use of the word challenge at the beginning of the paragraph suggests that geropsychology is still perceived to be in its beginning. A kind of test or trial rather than an established tradition. And again: “In our understanding, geropsychology can be characterized by the following defining features:

1. Geropsychology incorporates a life-span perspective on the ageing process;
2. It addresses mental and behavioral processes of ageing, and of older adults, both in research, and in applied fields of psychology;
3. It targets both at the improvement and consolidation of quality of life in later adulthood as well as the improvement of professional competence of psychologists in this field;
4. It includes assessment, education, intervention, and research in the field of ageing.

Expertise and knowledge of geropsychology will be necessary for all fields of applied psychology since ageing represents a transversal dimension that is important for many if not all domains of individual functioning. Geropsychology should thus be an integral part of all psychological training throughout Europe. Knowledge in geropsychology should be provided for different target groups”.

There does not seem to be a clear reference to the relationship between mental health and physical health, while the indications regarding specific training on the subject are rather broad and adopt hypothetical verbs. Finally, a link to a special issue on a topic of great impact and diffusion, “*Active Aging: A Global Goal*” [134], is provided, and a second link to another special issue entitled “*Geropsychology across Europe*” is also announced. On the other hand, on the website of the European Association of Clinical Psychology and Psychological Treatment [135], there is no explicit reference or subsections regarding clinical psychology for the elderly population.

In Italy, the most important psychological association (Italian Association of Psychology) [136] has established a working group named ‘Clinical Psychology of Aging’, which authored a seminal paper for the discipline in Italy, “The Italian Manifesto” [137]. This work likely serves as a comprehensive and authoritative document that outlines a vision, principles, and strategies for enhancing clinical psychology interventions for older adults in Italy, with the aim of improving their mental health and overall quality of life. It provides background information on the significance of addressing mental health issues in the elderly population, particularly within the context of clinical psychology. It may highlight the demographic shift towards an aging population globally and within Italy, underscoring the importance of tailored psychological interventions to address the unique needs of older adults. The authors underline gaps in existing approaches, and they suggest the need for a unified framework to guide clinical practice, research, and policy in this area, promoting active aging, enhancing the quality of life, preserving autonomy and dignity, and fostering social inclusion. Moreover, the authors propose a guiding framework based on evidence-based practices, theoretical perspectives (such as biopsychosocial models of aging), and cultural considerations relevant to the Italian context to improve access to mental health services for older adults to enhance interdisciplinary collaboration among healthcare professionals. At the time of writing, there is no link to the products of this group.

Moreover, in the Italian context, the Society of Psychology of Aging (SIPI) [138] is dedicated to the study and advancement of psychology related to aging in Italy. SIPI serves as a platform for researchers, psychologists, gerontologists, and other professionals interested in aging to come together, share knowledge, and collaborate on research and interventions aimed at addressing the unique psychological needs and challenges faced by older individuals. The society provides a forum for discussing research findings, emerging trends, and best practices in the field of aging psychology. SIPI organizes conferences, workshops, and seminars where experts in the field present their research findings, share insights, and engage in discussions on topics relevant to aging psychology. These events provide valuable opportunities for professionals to stay updated on the latest advancements in the field and network with colleagues who share similar interests. Furthermore, the Italian Association of Psychogeriatrics [139] is also active in this area, and the website provides various resources useful for research, training, and intervention related to this topic.

The Australian Clinical Psychology Association [140] serves the function of promoting culture, research, and education in clinical psychology. No clear references to geropsychology or clinical geropsychology are found on the site. The most significant reference to mental health from a psychological perspective in aging comes from the Australian Psychological Association [141] in one of the internal links we found on page [142] on psychological intervention policies for the elderly population. There are stated a series of key points, a series of political wills on which the Association is focusing to provide the necessary psychological support to the elderly population, a series of articles from the in-house journal InPsych that describe issues of close interest to the community, and ethical guidelines in the interaction with elderly clients.

In Asia, the most organized link dealing with gerontology issues is that of the Korean Gerontological Society (KGS) [143], which is a federated society of the Korean Association of Gerontology and Geriatrics (KAGG) [144]. In the pages devoted to KGS, we find general references to psychology along with many other disciplines. No reference is made to subspecialty geropsychology or clinical geropsychology.

In Africa, the most noteworthy resource based on the continental territory is the research and practice activity on aging named Aging and Development hosted by the African Population and Health Research Center [145]. In this case, several ongoing research projects are cited. The first primarily concerns the economic variable of the demographic dividend—the growth/loss in an economy that is the result of a change in the age structure of a country’s population—and aims to study the economic changes associated with demographic population shifts, as the average age has also increased in the African continent (Working Group on Aging and Achieving a First Demographic Dividend in Africa—APHRC). The second project is expected to advance the scientific and policy debate on later-life resilience globally in conditions of scarce resources, certainly a project that incorporates dimensions closely related to psychological interests: well-being, resilience to environmental obstacles, and extreme resource scarcity (Understanding Resilience in Later Life in a Low-Resource Setting—APHRC). The third described project involves a long-term survey initiated in 2013 with the objectives of “identifying priority national evidence needs on Kenya’s older population and forging a concerted country-level effort to foster the generation of evidence to address the needs” (Toward a Pilot Evidence Revolution on Ageing in Kenya—APHRC). There are no clear references to the mental health of older adults. Although these projects, at a development level, are only marginally attentive to the mental health needs of older adults, they have the merit of contextualizing research on the specific needs of individuals living in vastly different cultural, economic, educational, and health promotion service environments.

The emerging picture is, as expected, quite diverse. In the United States, there is a consolidated tradition of producing resources made available to all scholars and interested stakeholders. In other cases, there are more hesitant attempts or psychological topics are simply encompassed within the broader interests of gerontology, which, however, have markedly medical theoretical frameworks. In other cases, psychological subjects are intimately connected to the absolute scarcity of resources, and under such conditions, it is objectively highly demanding to contemplate mental health services if even basic physical health is scarcely regarded, absent, or accessible to a small part of the population [146,147,148,149,150].

## 5. Geropsychology: The Assessment

Assessments in geropsychology require a comprehensive and multidimensional approach. This approach acknowledges the diverse array of factors that influence the mental health and functioning of older adults, including biological, psychological, social, and environmental variables [151,152].

Firstly, biological factors play a crucial role in shaping the mental health and well-being of older adults. Age-related changes in brain structure and function can impact cognitive abilities, emotional regulation, and susceptibility to neurodegenerative disorders such as Alzheimer’s disease [153]. Additionally, the presence of chronic health conditions and the use of medications can further influence psychological functioning and may contribute to symptoms of depression, anxiety, or cognitive impairment. This type of investigation can be performed by gathering information about past and current medical conditions, medications, surgeries, and hospitalizations, assessing physical functioning, sensory impairments, mobility, and chronic health conditions [154,155].

Secondly, psychological factors encompass a broad spectrum of aspects, including personality traits, coping strategies, and emotional resilience, evaluating symptoms of depression, anxiety, and other mood disorders using standardized measures like the Geriatric Depression Scale (GDS) [156,157] and the Geriatric Anxiety Inventory (GAI) [151,158], assessing memory, attention, executive function, and language skills to detect cognitive decline or dementia, using standardized tests such as the Mini-Mental State Examination (MMSE) [159] or Montreal Cognitive Assessment (MoCA) [160,161,162]. Older adults may face unique psychological challenges such as adjusting to retirement, dealing with grief and loss, or navigating existential questions related to mortality. Moreover, past life experiences and trauma can significantly shape an individual’s psychological well-being in later life, underscoring the importance of considering the lifespan perspective in assessment.

Moreover, intraindividual and interindividual variability should also be considered. Fluctuations in a person’s cognitive, emotional, or physical performance over time can indicate the early stages of cognitive decline or other health issues. Understanding intraindividual variability helps in identifying the factors that contribute to these fluctuations and developing personalized interventions to maintain or improve functioning [163]. Interindividual variability emphasizes the diversity of aging experiences across the population. It can be attributed to a range of factors, including genetics, lifestyle choices, social environment, education, and overall health. Interindividual variability underscores the importance of not treating the elderly population as a homogeneous group [164].

Social factors also play a critical role in the mental health and functioning of older adults. Social support networks, relationships with family and friends, and participation in meaningful activities can serve as protective factors against loneliness, depression, and cognitive decline and can promote empathy [165,166,167]. Empathy, especially cognitive empathy, helps older adults sustain meaningful relationships, which in turn supports their mental and emotional well-being. Conversely, social isolation, bereavement, or changes in living arrangements can increase vulnerability to mental health problems, highlighting the importance of assessing the social context in which older adults live. The availability and quality of social support networks, including family, friends, and community resources, are assessed not only by evaluating the quality of relationships and the impact of social interactions on mental health and well-being but also with the engagement in meaningful activities, hobbies, volunteering, and social participation [168,169].

Particularly within the domain of social functioning, it is crucial to include evaluations of socio-cognitive functioning and theory of mind, as these aspects play a significant role in everyday life functioning for older adults. Socio-cognitive functioning encompasses the processes that allow individuals to interpret and respond to social cues, which are essential for maintaining relationships and effectively navigating social environments [170]. Theory of mind, the ability to understand others’ mental states, is particularly relevant as it underpins empathy, social decision-making, and effective communication. These cognitive faculties are often subject to decline in older adults, impacting their social interactions and overall quality of life. Therefore, incorporating these areas into geropsychological assessments can provide a more comprehensive understanding of an individual’s social capabilities and challenges, ultimately guiding more effective interventions and support strategies [171].

Furthermore, environmental variables encompass the broader societal and environmental factors that impact the lives of older adults. Access to healthcare services, socioeconomic status, housing conditions, and community resources can profoundly influence mental health outcomes and quality of life in later adulthood. Environmental stressors such as financial strain, discrimination, or ageism can exacerbate existing mental health issues and contribute to disparities in access to care [172,173]. Moreover, environmental variables such as the safety, accessibility, and suitability of the individual’s living arrangements, including housing conditions and adaptations for aging in place, could be considered. All these conditions determine the individual’s mobility status, fall risk, and safety awareness within their environment.

Additionally, cognitive reserve, which refers to the mind’s resilience to neuropathological damage, is a critical factor. The Cognitive Reserve Index Questionnaire (CRI-q) can be used to assess an individual’s cognitive reserve. This tool measures various aspects of cognitive reserve, including education, occupational complexity, and engagement in cognitively stimulating activities. Higher cognitive reserve is associated with a better ability to cope with brain changes and maintain cognitive function in older age [174,175,176].

Lastly, functional assessment testing can provide valuable information about home care activities. Functional assessment is an effective way to objectively document a patient’s functional status, progress through the episode of care, and justify homebound status through the evaluation of Activities of Daily Living (ADLs) [177] to estimate the individual’s ability to perform basic self-care tasks independently, such as bathing, dressing, eating, and toileting, and the Instrumental Activities of Daily Living (IADLs) [178] to ascertain the higher-level functional abilities related to household chores, meal preparation, medication management, and financial management.

In light of this, a comprehensive and multidimensional approach to assessment in geropsychology is essential for understanding the diverse array of factors that shape the mental health and functioning of older adults. By considering the interplay of biological, psychological, social, and environmental variables, mental health professionals can develop more nuanced assessments and interventions tailored to the unique needs of older adults, ultimately promoting optimal well-being and quality of life in later life stages. This personalized approach not only enhances the effectiveness of interventions but also promotes a sense of autonomy and dignity for older adults. Accepting this perspective, the Integrated Care for Older People (ICOPE) [179,180] was created. It is a comprehensive approach designed to address the multifaceted needs of elderly individuals, focusing on person-centered assessment and pathways within primary care settings. The ICOPE screening tool is currently available in different languages: Arabic, Chinese, Japanese, Portuguese, Russian, Spanish, Vietnamese and Indonesian. The Italian version is newly published, edited by Solimando, Barbagallo, and Veronese (at the University of Palermo), and revised by Bosco and Lopez (at the University of Bari). The ICOPE approach aims to integrate seamlessly into primary care settings for older individuals, structured around five key steps. Step 1 involves screening participants to identify potential declines across the six domains of intrinsic capacity. Step 2 entails conducting a thorough assessment of those identified during screening, examining deficits in intrinsic capacity alongside their underlying conditions and physical and social environments. Step 3 focuses on crafting a personalized care plan, considering declines in intrinsic capacity, concurrent diseases, socioenvironmental factors, and, critically, the older person’s goals and preferences. ICOPE recommends regular monitoring of intrinsic capacity every 6 months, even for individuals who show no initial declines during screening. Similarly, ongoing monitoring of the personalized care plan proposed post-assessment (step 4) is advised. Step 5, a cross-cutting component, emphasizes community involvement and caregiver support to facilitate the implementation of the preceding steps.

ICOPE recognizes the unique challenges faced by aging populations and aims to promote holistic care that considers not only medical needs but also social, psychological, and functional aspects, highlighting its emphasis on holistic assessment, multidimensional interventions, and collaborative care coordination. The ICOPE assessment process emphasizes the importance of tailoring interventions to individual preferences, values, and circumstances. Drawing upon evidence-based practices and interdisciplinary collaboration, ICOPE represents a paradigm shift towards proactive and preventive care for older populations. ICOPE involves evaluating various domains of health and well-being, including physical function, cognition, mental health, social support, and environmental factors. By conducting a thorough assessment, healthcare providers can identify areas of strength and areas that may require intervention, allowing for targeted and personalized care strategies. ICOPE also emphasizes the importance of collaboration and coordination among healthcare professionals, caregivers, and community resources. Integrated care teams, comprising physicians, nurses, social workers, and other specialists, work together to address the diverse needs of older adults comprehensively. Additionally, partnerships with community organizations and support services help ensure continuity of care and access to resources beyond the healthcare setting.

By identifying risk factors and early signs of decline, healthcare providers can implement strategies to prevent or delay functional decline, frailty, and other age-related conditions. This proactive approach not only improves individual outcomes but also reduces healthcare costs associated with managing preventable complications. The ICOPE framework provides policymakers and program managers with a comprehensive tool for the system-level analysis required to develop the necessary action plans.

It is right to mention the DSM-5^®^ Pocket Guide for Elder Mental Health [181]. It is a concise and practical reference tool for mental health professionals working with older adults. It distills key information from the Diagnostic and Statistical Manual of Mental Disorders, Fifth Edition (DSM-5), tailored to the unique needs and considerations of elderly individuals. The guide likely introduces the field of geriatric mental health, highlighting the prevalence of mental health disorders among older adults and the importance of accurate diagnosis and effective treatment. It includes condensed diagnostic criteria for a range of mental health disorders commonly encountered in older adults, such as neurocognitive disorders (e.g., Alzheimer’s disease, vascular dementia), mood disorders (e.g., major depressive disorder, bipolar disorder), anxiety disorders (e.g., generalized anxiety disorder, panic disorder), and psychotic disorders (e.g., schizophrenia). Moreover, the guide makes some age-related considerations discussing how mental health disorders may present differently in older adults compared to younger individuals, including variations in symptomatology, comorbidity patterns, and treatment response. It emphasizes the importance of considering age-related factors in the assessment and diagnosis process. Its strengths include the assessment tools and the treatment guidelines. It includes brief screening tools and assessment instruments specifically designed for use with older adults, such as the MMSE for cognitive impairment or the GDS for depression screening, and it offers concise treatment recommendations and guidelines for managing mental health disorders in older adults. This includes pharmacological interventions, psychotherapy approaches, and non-pharmacological interventions tailored to the unique needs and preferences of elderly individuals. Also, this guide discusses practical considerations for providing holistic care to older adults with mental health disorders, including interdisciplinary collaboration, caregiver support, and strategies for addressing psychosocial needs.

## 6. Geropsychology Training Programs

We have said that geropsychology is a field that serves, values, and advocates for older adults. In a previous paragraph, we reported that the societies of Clinical Geropsychology can be charged to the most accredited training programs. The APA Guidelines for Psychological Practice With Older Adults [129], since its first release, intended to assist psychologists in evaluating their own readiness for working with older adults and in seeking and using appropriate education and training to increase their knowledge, skills, and experience relevant to this area of practice, with the specific goals of providing practitioners with (a) a frame of reference for engaging in clinical work with older adults and (b) basic information and further references in the areas of attitudes, general aspects of aging, clinical issues, assessment, intervention, consultation, professional issues, and continuing education and training relative to work with this group. Furthermore, APA created the APA Careers in Aging Roadmap, a comprehensive guide for psychologists seeking to make a meaningful impact in the field of geropsychology, offering practical advice and resources to navigate the diverse opportunities and challenges in this rewarding area of practice. It outlines a step-by-step educational roadmap to help undergraduate and graduate students find careers in aging. It includes specific questions to consider at each educational level, actions to take to be prepared and positioned to embark on a career in aging, resources (directories of programs in psychology and gerontology, academic resources, preparing for graduate school, networking, and how to prepare for a job search), career profiles, and interdisciplinary careers that intersect with aging including those in engineering, business, law and policy, and biological and health sciences. It can be considered a strategic guide for psychologists interested in pursuing careers focused on aging-related issues, including specialized coursework, training programs, or degrees focused on geropsychology aging studies or related disciplines, to understand the biological, psychological, social, and cultural aspects of aging, as well as the unique challenges and opportunities associated with aging populations; opportunity for completing practicum or internship placements in settings such as nursing homes, assisted living facilities, hospitals, or community mental health centers; interdisciplinary collaboration and research opportunities, for career advantages.

The Geriatric Workforce Enhancement Programs (GWEPs) also provide training across the country. Funded by the Health Resources and Services Administration (HRSA), the GWEP’s aims to improve health outcomes for older adults by developing a healthcare workforce that maximizes patient and family engagement and by integrating geriatrics and primary care. The goals of this program are as follows: (1) to educate and train the primary care and geriatrics workforce to care for older adults in integrated geriatrics and primary care models, and (2) to partner with community-based organizations (CBOs) to address gaps in healthcare for older adults, promote age-friendly health systems and dementia-friendly communities, and address the social determinants of health.

Moreover, the Council of Professional Geropsychology Training Programs (CoPGTP) is dedicated to promoting training in geropsychology on four broad levels: graduate, pre-doctoral internship, post-doctoral fellowship programs, and post-licensure.

All these trainings are based on the Pikes Peak Model for Training in Professional Geropsychology [182] regarding attitude (awareness and belief about the personal disposition to aging), knowledge (understanding the processes of aging), and skill competencies (to apply ethical and legal standards, with particular attention to aging-specific issues) for practice in professional geropsychology. It is structured as a self-evaluation of learning needs to assist psychologists in evaluating their own scope of competence for working with older adults. By integrating these attitudes, knowledge, and skill competencies into their practice, professionals in geropsychology can provide comprehensive and effective care for older adults, promoting their mental health and well-being.

In 2002, Qualls and colleagues [92] stated that a large majority of participants in an APA survey regarding psychological practice managed clinical work with elderly people, but less than one-third of them followed graduate coursework on the topic in the past, and only one fifth have an internship experience with seniors.

In 20 years, will something change? In 2019, Moye [183] and colleagues reported the results of another survey, the American Psychological Association’s Center for Workforce Studies survey of psychologists, with a focus on older adults, where doctoral psychologists were identified through state licensing boards. Less than 2% (participants = 4109) of licensed doctoral psychologists labeled geropsychology as their specialty area, although more than one-third of the total reported interacting for clinical intervention with older adults, often from other specialties such as rehabilitation psychology, clinical neuropsychology, and clinical health psychology. Moreover, these surveys are conducted in the USA, which is less known regarding other countries, but it is not likely to suggest substantially different scenarios. Everybody knows that the health welfare systems have been significantly worsened by the global pandemic; nonetheless, the health needs of elderly people should also be addressed, with specific attention to mental health. In turn, the training program in clinical psychology (but also those in experimental, cognitive, and social psychology) should be rethought in this context.

## 7. Discussion

Geropsychology is a field of psychology dedicated to utilizing psychological knowledge and methodologies to comprehend and assist older individuals and their families in preserving well-being, surmounting challenges, and realizing their fullest potential as they progress through later stages of life. Geropsychology encompasses various fields of psychology due to the multifaceted nature of aging and its impact on individuals, such as developmental, clinical, social, health psychology and neuropsychology, and psychometrics. So, the study of geropsychology includes the understanding of the psychological changes and transitions that occur across the lifespan, involving those specific to aging; a focus on diagnosing, assessing, measuring, and treating mental health issues that commonly affect older adults, such as depression, anxiety, and dementia; the investigation of the social factors that influence aging, such as social support, relationships loneliness, social isolation, ageism, and societal attitudes towards older adults [184]. Geropsychology significantly intersects with other disciplines, particularly medicine, because mental health in aging necessarily intersects with organic health issues, chronic conditions and disabilities, age-related changes, and neurological disorders such as Alzheimer’s disease and Parkinson’s disease. Overall, geropsychology addresses the diverse needs and challenges of older adults, highlighting the importance of a multidisciplinary approach to promoting mental health and well-being in later life, benefiting from attention at the policy and legislative levels to enhance it.

The integration of research findings into evidence-based interventions and service programming is essential for promoting health and reducing the burden of disease in communities. Collaboration among researchers, practitioners, policymakers, and community stakeholders is crucial for developing and implementing effective strategies for health promotion in aging [185].

The results coming from the research could be incorporated into policy agendas and decision-making processes so that stakeholders could work together to create environments that support the mental health and well-being of older adults, ultimately promoting healthy aging and enhancing the quality of life for older populations [186].

This work could be considered as a sort of memorandum, a strong interdisciplinary and cross-national connotation to prioritize mental health through health promotion and disease prevention, also supporting equitable access to mental health services and integrating mental health into primary care, encouraging continued education and skill-building opportunities for older adults to foster cognitive vitality and maintain mental acuity throughout the aging process, to foster social connectedness, supporting caregivers and age-friendly community initiatives and policies that promote social inclusion, accessibility, and opportunities for meaningful engagement for older adults.

Fostering the collaboration among policymakers, healthcare providers, researchers, community organizations, and older adults themselves, the chance to develop comprehensive and holistic approaches to promoting mental health and well-being in later life will become a priority. This change of pace has practical implications for the healthcare systems. A significant portion of healthcare expenditure in the most developed countries is dedicated to the care of elderly individuals [187]. Promoting primary prevention from a psychological perspective can help prevent the most negative effects of illness and the so-called “revolving door” effect [188], a situation where patients repeatedly come back to the same service with the same health issues. It would, therefore, be advisable to keep elderly individuals away from hospitals for as long as possible.

## 8. Strengths and Limitations

This viewpoint paper presents some strengths and limitations. First, it represents a comprehensive understanding of geropsychology, integrating knowledge from diverse disciplines to address the complex needs of older adults and intersecting significantly with fields like medicine, addressing mental health issues alongside organic health problems, chronic conditions, and neurological disorders such as Alzheimer’s and Parkinson’s diseases. Moreover, it highlights the importance of a multidisciplinary approach, advocating for attention at policy and legislative levels to enhance mental health and well-being in later life. Notwithstanding this, some limitations cannot be disregarded regarding the lack of empirical validation and the presence of subjectivity due to the authors’ perspectives and biases.

## 9. Future Perspectives

Moving forward, research in geropsychology should explore cognitive changes associated with aging and interventions to maintain or enhance cognitive functioning; factors contributing to psychological resilience in older adults, including social support and coping mechanisms; the role of technology in supporting mental health and quality of life in older adults; and social and environmental factors influencing mental health, such as age-friendly community initiatives and intergenerational relationships.

More specifically, future lines of research in this field could investigate the following: (a) cognitive changes associated with aging and developing interventions to maintain or enhance cognitive functioning in older adults; (b) factors that contribute to psychological resilience in older adults, including coping mechanisms, social support networks, and personal strengths; (c) the role of technology in supporting older adults’ mental health and quality of life studying the use of digital health tools, telemedicine, virtual reality interventions, and assistive technologies for older adults with cognitive or physical impairments; (d) the impact of social and environmental factors on older adults’ mental health and well-being, including research on age-friendly community initiatives, urban design features that support aging in place, and social inclusion programs for older adults; (e) the benefits of intergenerational interactions and relationships for older adults’ mental health and social connectedness, exploring the effects of intergenerational programs, mentoring initiatives, and family dynamics on aging outcomes.

## 10. Conclusions

In conclusion, integrating support services (e.g., counseling, psychotherapy), educational programs to promote awareness about aging and reduce associated stigma, cognitive stimulation programs to maintain and enhance cognitive functions, home care services, and community support facilitates access to care. For the caregivers, primary prevention programs and active aging strategies should clearly recognize the importance of caring for older persons and the critical need to take proactive measures to address the needs of the elderly. These efforts are essential in promoting healthy aging and ensuring that older adults have the right to live with dignity, independence, and quality of life worldwide [189]. It is necessary to educate the public about the importance of adopting and maintaining healthy habits throughout life. Public health initiatives should focus on raising awareness about the connection between lifestyle choices and cognitive health, encouraging behaviors that promote long-term brain health. By increasing awareness and promoting proactive lifestyle changes, it is possible to reduce the prevalence of dementia and improve the quality of life for older adults.

## Data Availability

No new data were created or analyzed in this study. Data sharing is not applicable to this article.
